# The Effects of Cetirizine on P-glycoprotein Expression and Function *In vitro* and *In situ*

**DOI:** 10.15171/apb.2016.017

**Published:** 2016-03-17

**Authors:** Mehran Mesgari Abbasi, Hadi Valizadeh, Hamed Hamishekar, Leila Mohammadnejad, Parvin Zakeri-Milani

**Affiliations:** ^1^ Drug Applied Research Center, Tabriz University of Medical Sciences, Tabriz, Iran.; ^2^ Students Research Committee, Tabriz University of Medical Sciences, Tabriz, Iran.; ^3^ Immunology Research Center, Tabriz University of Medical Sciences, Tabriz, Iran.; ^4^ Liver and Gastrointestinal Diseases Research Center and Faculty of Pharmacy, Tabriz University of Medical Sciences, Tabriz, Iran.

**Keywords:** Cetirizine, Digoxin, Intestinal permeability, P-glycoprotein

## Abstract

***Purpose:*** P-glycoprotein (P-gp) plays a major role in oral absorption of drugs. Induction or inhibition of P-gp by drugs contributes to variability of its transport activity and often results in clinically relevant drug-drug interactions. The purpose of this study was to investigate the effect of cetirizine, a second generation H_1_ antihistamine, on P-gp function and expression *in vitro *and *in situ*.

***Methods:*** The *in-vitro* rhodamin-123 (Rho123) efflux assay in Caco-2 cells was used to study the effect of cetirizine on P-gp function. Western blot analysis was used for surveying the effect of cetirizine on expression of P-gp in Caco-2 cells. Rat in situ single-pass intestinal permeability technique was used to calculate the intestinal permeability of a known P-gp substrate (digoxin) in the presence of cetirizine. The amounts of digoxin and cetirizine in intestinal perfusion samples were analyzed using a HPLC method.

***Results:*** The results showed significant increase in Rho123 uptake (P < 0.05) and also P-gp band intensity decrease in cetirizine-treated cells *in vitro*. Furthermore the intestinal permeability of digoxin was also increased significantly in the presence of cetirizine (P < 0.01).

***Conclusion:*** Therefore it is concluded that cetirizine is a P-gp inhibitor and this should be considered in co administration of cetrizine with other P-gp substrate drugs. Further investigations are required to confirm our results and to determine the mechanism underlying P-gp inhibition by cetirizine.

## Introduction

Currently, most drugs used in clinical practice are administered orally and must be adequately and consistently absorbed to achieve successful therapy. Appropriate regulation of drug delivery to the body, including intestinal absorption, hepatic metabolism, renal excretion, and transport into the tissues, is important to ensure suitable therapeutic efﬁcacy. Maintaining appropriate serum concentration of drugs is also necessary to exert their beneﬁcial effects and to prevent unexpected adverse effects, especially for drugs with narrow therapeutic index. Furthermore, the increasing potential of clinical drug–drug, herb–drug, and food-drug interactions resulted from developing new drugs and increasing wide-use of drugs and herbal drugs have been noticed. In addition to other mechanisms, membrane transporters are the most important factors involved in the mentioned interactions and they are major barriers limiting oral drug delivery.^1,2^

P-glycoprotein (P-gp) is the most important membrane transporter which is responsible for secreting (active efflux) passively diffused drugs and xenobiotics out of the cell.^2,3^ P-gp is an ATP binding cassette protein and plays a major role in drug absorption and distribution due to its abundant expression on several barrier epithelia (including the epithelial cells of intestine, kidney, liver, brain, testis, adrenal gland, placenta, and bile canalicula) and its broad substrate speciﬁcity.^3^ Some drugs, foods, and chemicals may be substrates of P-gp. The transport activity of P-gp can be induced or inhibited *in vivo* by a variety of drugs with different structures, such as verapamil, rifampicin, erythromycin, ketoconazole, and cyclosporine^4,5^ which may affect the absorption of drugs themselves and the concomitantly used drugs. Induction (enhancing P-gp activity) or inhibition (impairing P-gp-mediated efﬂux) of P-gp by drugs or other xenobiotics contributes to variability of its transport activity and often results in clinically relevant interactions. Therefore, P-gp-related interactions have important clinical impacts and it is critical to understand which drugs are inducers or inhibitors of P-gp to minimize or avoid adverse interactions.^3,6^

**Figure 1:**
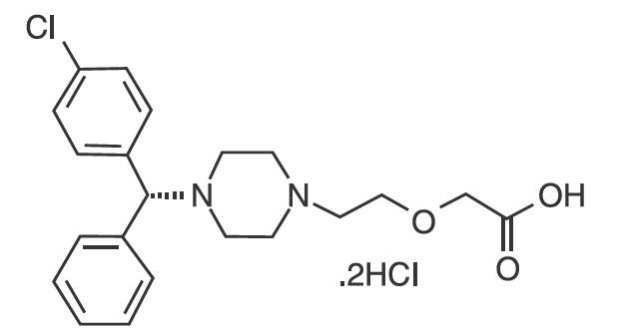
Chemical structure of cetirizine.^10^

Cetirizine, a member of the second generation H_1_ antihistamines, is chemically known as (RS)-2-[2-[4-(4-chlorophenyl) phenylmethyl] piperazin-1-yl] ethoxy] acetic acid dihydrochloride (Figure 1). Cetirizine is a piperazine derivative and is marketed as a racemic mixture containing both levocetirizine and dextrocetirizine. It is a long-acting non-sedative antihistamine and an antagonist of H_1_-receptor. Cetirizine di-hydrochloride is used for symptomatic treatment of allergic conditions including seasonal allergic rhinitis and chronic urticarial.^7-9^

Therefore the purpose of this study was to investigate the effect of cetirizine treatment on the P-gp function and its expression both *in vitro* and *in situ*. In addition, the potential role of digoxin, a typical substrate of P-gp, in intestinal effective permeability (P_eff_) of cetirizine was also studied.

## Materials and Methods

### Materials

Anti-P-gp mouse monoclonal antibody (ab80594) and rabbit polyclonal antibody (Ab) to beta actin (ab16039) were purchased from Abcam (Cambridge, UK). Horseradish peroxidase (HRP) conjugated anti-mouse Immunoglobulin (IgG) (AP8036) and anti-rabbit IgG (AP7181) were purchased from Razi Biotech (Karaj, Iran). Rhodamin-123 (Rho123), digoxin, verapamil, cetirizine, 3-(4,5-dime-thylthiazol-2-thiazolyl)-2,5-diphenyl-tetrazoliumbromide (MTT), penicillin-streptomycin, protease inhibitor cocktail and nonfat-dried milk bovine were purchased from Sigma–Aldrich (St. Louis, MO, USA). Dulbecco's modified eagle medium (DMEM), trypsin-EDTA (0.25%), fetal calf serum (FCS) were purchased from Gibco (Carlsbad, CA, USA). Tissue culture flasks and other disposable cell culture items were purchased from TPP Co. (Trasadingen, Switzerland). Enhanced chemiluminescence (ECL) western blotting detection kit, medical X-ray film, pre-stained protein ladder (10-250 kDa), and protein assay kit were purchased from Amersham (GE Healthcare, Chalfont St. Giles, UK), Fuji (Tokyo, Japan), Cinagen, and Pars Azmoon (Tehran, Iran), respectively. All other chemicals were purchased from Merck Co. (Darmstadt, Germany).

### Cell Culture

Caco-2 cells were obtained from national cell bank of Iran (Pasteur Institute, Tehran, Iran) and were routinely cultured in Dulbecco’s modified Eagle’s medium (DMEM) containing 10% fetal bovine serum, 10,000 I.U./mL Penicillin, and 10,000 μg/mL Streptomycin. Cells were maintained in an incubator at 37°C with a humidiﬁed atmosphere of 5% CO_2_ in a CO_2_ incubator (Memmert, Schwabach, Germany). The culture medium was changed 2-3 times per week. After 2-3 weeks culture and differentiation of Caco-2 into intestinal-like cells, the cells were detached from the culture flask by addition of 0.25% trypsin-EDTA. Cells were then seeded at the needed density for performing the following tests.

### Cytotoxicity study (MTT assay)

The MTT assay was used to determine the cytotoxicity of 0.2, 10, and 100 µM cetirizine. The Caco-2 cells were seeded into 96-well plates at a density of 15 × 10^3^ cells per well. After 24 h, the medium was replaced with 200 µL per well of cetirizine different concentrations. After 24 h of incubation, MTT solution (2 mg/mL final concentration) was added to each well and incubated for 4 h at 37 °C in a CO_2_ incubator. The MTT solution was removed and the resulting formazan crystals were solubilized with 200 µL/well of dimethyl-sulfoxide (DMSO) and 25 µL/well Sorensen buffer. The optical densities (ODs) were measured with an enzyme linked immune-sorbent assay (ELISA) plate reader (Statfax-2100, Awareness, Palm City, FL, USA) at 570 nm with background subtraction at 630 nm. The following formula was used for calculating percentage of cell viability.

(1) $$\text{Cell viability \%}=\frac{\text{OD value of test}}{\text{OD value of control}}\;\times 100$$

The mean ± standard deviation (SD) was calculated for each concentration and analyzed statistically.

### Rho123 efflux assay 

P-gp function study in Caco-2 cells was realized by the measurement of intracellular accumulation of Rho123, a well-known P-gp substrate, which is inversely proportional to P-gp activity. Caco-2 cells were seeded in 24‏-well plates and allowed to attach for 24 h. Then, the old medium was removed and cells were washed with PBS. New culture media containing 100 µM cetirizine and 300 µM verapamil, as P-gp inhibitor, were added to separate wells and left for another 24 h. After that, the old medium was removed and cells were washed three times with PBS. Rho123 solution [DMEM containing 10 mM HEPES (pH = 7.4) and 5 μM Rho123] was added and incubated for 3 h at 37°C, followed by three washes with ice-cold PBS (pH = 7.4). Cells were lysed in 1 % Triton X-100 and centrifuged at 1500 g for 10 min (3-18k, Sigma). Supernatant was used to measure the Rh123 and the total protein contents. The cellular accumulation of Rho123 was determined quantitatively by fluorescence spectrophotometry (λ_ex_ = 485 nm, λ_em_ = 532 nm) (FP-750, Jasco, Japan) and normalized by the protein content of each sample determined by the protein assay kit (Pars Azmoon, Iran).^11-13^

### Western blot 

Caco-2 cells were seeded to a 6 well-plate in the density of 10^6^ cells per well and treated for 48 h with the culture medium (as control), culture medium containing 0.2, 10, and 100 µM cetirizine, and 300 µM verapamil, as P-gp inhibitor. Then, solutions were removed and cells were washed with PBS, and incubated for 5 min with trypsin/EDTA 0.25 % in 37 °C. The detached cells were washed twice with PBS. Lysis buffer (Triton X-100 50 mM, NaCl 150 mM, EDTA 5 mM, 1 % protease inhibitor cocktail, tris-HCl, pH=7.4‏( was added and cell suspension was centrifuged at 1500 g for 5 min. Proteins were separated through sodium dodecyl sulfate-polyacrylamide 12.5‏ % running gel (SDS-PAGE) electrophoresis (80 V, 120‏ min). The gel was electro blotted to a poly-vinylidene di-fluoride (PVDF) membrane using a semi-dry western blotting system (Bio-Rad, Hercules, CA, USA). All membranes were blocked for nonspecific binding by incubation in 3% nonfat-dried milk for 1 h at room temperature and the membrane was washed 3 times with Tris buffered saline (TBS) with 0.1% Tween 20. Then, the membranes were incubated overnight with mouse monoclonal anti-P-gp antibody (1/1000 in TBS). After washing with TBS, the membrane was incubated with HRP-conjugated rabbit anti-mouse secondary antibody for 2 h. Membrane was washed and the proteins were then detected using an ECL kit. The proteins were visualized by exposing the membrane to a medical x-ray film (Fuji, Japan) for 5 min in a dark room and scanned using a bio-imaging analyzer (Bio-Rad, USA). β-actin was the internal standard, and was detected using rabbit polyclonal anti-β-actin as primary antibody and HRP-conjugated goat anti-rabbit IgG as secondary antibody. P-gp expression is presented as the ratio of P-gp band intensity to β -actin band intensity in the same blot run (P-gp/ β –actin).^14^

### In situ single-pass intestinal permeability study

#### Animals

Male Sprague–Dawley rats (200–250 g) were supplied by Pasteur Institute of Iran. The animals were kept under 22 ± 2°C with a 12/12 light/dark cycle, and 55 – 60% relative humidity. Prior to experimentation, rats were fasted for 12-16 h (water *ad libitum*). All procedures were conducted in accordance with the humanity and animal ethic protocols. The animal study protocols were approved by the research ethical committee of Tabriz University of Medical Sciences (Ref No: TBZMED.REC.1394.378).

#### Single-pass intestinal perfusion (SPIP)

The animals were anesthetized with an intra-peritoneal injection of thiopantal sodium (60 mg/kg). A midline abdominal incision of 3-4 cm was made and approximately 10 cm of the jejunum segment of the intestine was isolated and cannulated at both ends with polypropylene tubes. The exposed segment was kept moist with body tempered saline. At first, the segment was rinsed with 37℃ normal saline to wash and clear the segment, then PBS (pH = 7.4) containing 50 mg/l phenol red without drug (blank solution) was pumped through the segment at a constant flow rate of 0.2 ml/min (Q_in_) through a volumetric infusion pump (Argus Medical AG, Switzerland). Blank perfused solution was collected at the outlet and used to prepare cetirizine and digoxin calibrator solutions and also for stability studies.^15,16^ After reaching steady state, perfusates were quantitatively collected for every 10 min (2ml) lasting 90 min (9 samples). Water absorption and secretion and other changes during the perfusion may cause errors in the calculated permeability values, phenol red, therefore, was added at a concentration of 50 mg/L as a non-absorbable marker to correct the results. Digoxin (20 µM in blank solution), Verapamil as a typical P-gp inhibitor (in blank solution with digoxin 20 µM), and different concentrations of cetirizine (0.2, 10, and 100 µM in blank solution with digoxin 20 µM/L) were administrated as described above (n = 3) for each drug and each concentration).^15,16^ At the end of procedure, the length of the segment was measured (cm) and the animal was euthanized. Samples were stored at -70°C (ultra-low temperature freezer, Jal Tajhiz Production, Karaj, Iran), until analysis. The concentrations of phenol red in perfused (outlet) samples were measured at 560 nm using an UV-VIS spectrophotometer (Ultra-spec 2000, Pharmacia, Pfizer, New York, NY, USA).^17^ Digoxin and cetirizine amount in outlet samples were detected by HPLC method.

#### HPLC analysis of digoxin and cetirizine in intestinal perfused samples

**Figure 2:**
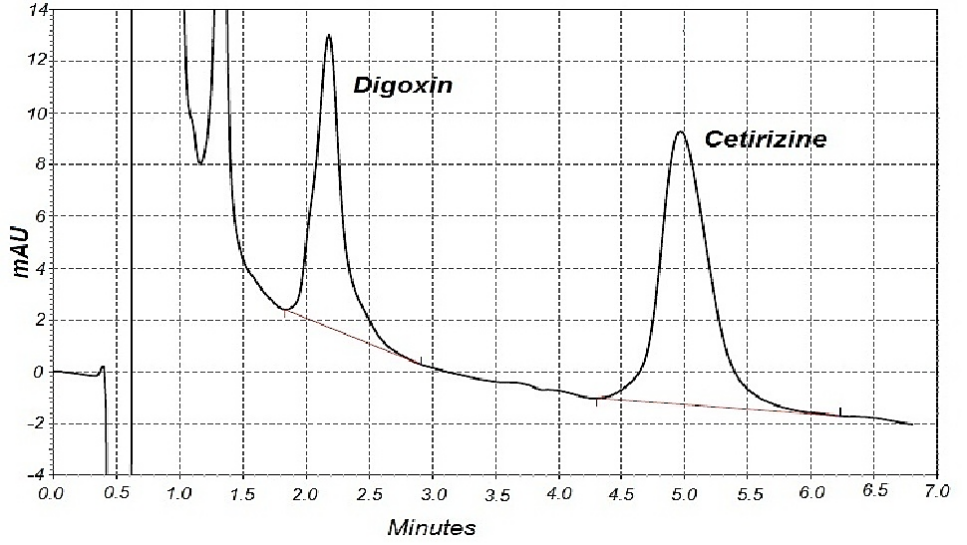
A representative HPLC chromatogram of cetirizine (10 µM) and digoxin (20 µM) in intestinal perfused sample.

The mobile phase for digoxin and cetirizine analysis was 35% (v/v) acetonitrile in water which was filtered through sintered glass filter P5 (1.0-1.6 μ, Winteg, Germany) and degassed in a sonicator. The mobile phase was pumped in isocratic mode at a flow rate of 2 ml/min at ambient temperature. UV detection was accomplished at 218 nm and samples of 20 μl were injected using Hamilton injector syringe (Hamilton, Switzerland) onto the column (Knauer- 15VE081ESJ - 150X4.6 mm with precolumn- Eurospher 100-5 C8, Berlin-Germany).^15^ The high performance liquid chromatography (HPLC) system was composed of Smartline manager 5000, Smartline UV detector 2600, and Smartline pump 1000 (Knauer Advanced Scientific Instruments, Berlin, Germany). Figure 2 shows a representative HPLC chromatogram of analyzed samples.

#### Data analysis

P_eff_ values were calculated from the steady-state concentrations of compounds in the perfusate collected from the outlet tubing. Steady state was confirmed by the ratio of the outlet to inlet concentrations (corrected for water transport) versus time.^15-17^ Corrected C_out_ (outlet concentration of the drug) was calculated from the following equation.^18^

(2) $${\text{C}}_{\text{out }\left(\text{corr}\right)}={\text{C}}_{\text{out}}\times \frac{\text{Concentration of phenol red in }\left({\text{CPR}}_{\text{in}}\right)}{\text{Concentration of phenol red out }\left({\text{CPR}}_{\text{out}}\right)}$$

Where C_out (corr)_ is corrected outlet concentration of the drug, C_out_ is outlet concentration of the drug, CPR_in_ is concentration of phenol red entering the intestinal segment and CPR_out_ is concentration of phenol red leaving the intestinal segment. Calculations were based on outlet perfusate steady state concentrations achieved after the selected time points. The steady-state intestinal P_eff_ was calculated according to following equation:

(3) $${\text{P}}_{\text{eff}}=\frac{-{\text{Q}}_{\text{in}}\;\times \text{ln }\left({\text{C}}_{\text{out}}/{\text{C}}_{\text{in}}\right)}{2\;\times 60\text{πrl}}$$

P_eff_ represents effective permeability (cm/s), Q_in_ represents the perfusion rate (0.2 ml/min), C_in_ and C_out_ represent the concentrations of the test drug entering and leaving the segment respectively, r is the radius of the intestinal segment (0.18 cm), and l is the length of the intestinal segment (cm).

### Statistical analysis

Data were presented as the mean ±SD. Statistical analyses were carried out using one-way analysis of variance (ANOVA) followed by Tukey’s multiple comparison test and the differences between two groups were determined using the unpaired t-test. Statistical tests were performed with SPSS13.0 (SPSS Inc., Chicago, IL, USA), where p < 0.05 and p < 0.01 were considered to be statistically signiﬁcant.

## Results

Preliminary experiments showed that no considerable adsorption of compounds on the tubing and syringe took place and the stock and working standard solutions of cetirizine in water was stable during the experiments.^9^

### Cytotoxicity study (MTT assay)

**Figure 3:**
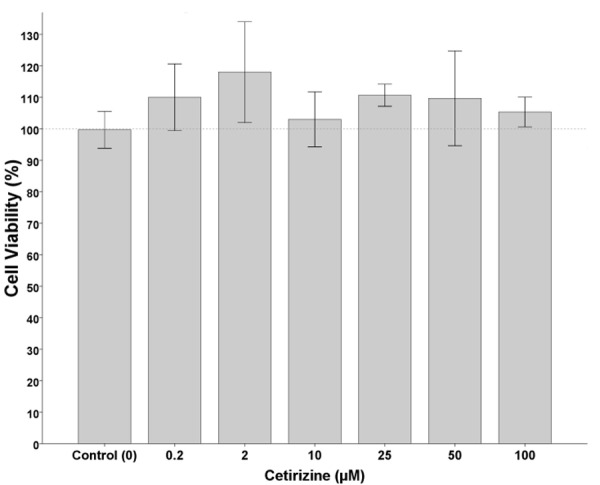
Effects of different concentrations of cetirizine on Caco-2 cells viability. Bars show mean ± SD of at least 3 measurements. * p < 0.05 was considered as significance level.

The cytotoxicity on cells was detected using MTT assay. Cetirizine, verapamil, and digoxin solutions showed no cytotoxicity on Caco-2 cells under the maximum dosage (100, 300, and 20 µM, respectively) (Figure 3).

### Rho123 efflux assay

**Figure 4:**
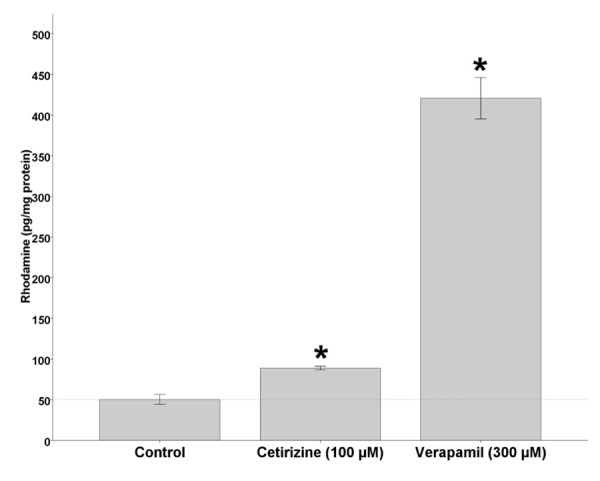
Effects of cetirizine and verapamil treatments on the intracellular accumulation of Rho123 in Caco-2 cells. Bars show mean ± SD of at least 4 measurements. * p < 0.05 was considered as significance level.

To assess the impact of cetirizine treatment on the function of P-gp as an efﬂux pump *in vitro*, we examined the P-gp-mediated Rho123 transport in Caco-2 cells treated with cetirizine using verapamil as a P-gp inhibitor. As shown in Figure 4, 100 µM cetirizine signiﬁcantly increased the intracellular accumulation of Rho123 in Caco-2 cells (p < 0.05). The mean intracellular concentration of Rho123 in control cells was 50.2 ± 6.0, while in cetirizine and verapamil treated cells were 88.8 ± 2.3 and 420.6 ± 25.4 pg/mg protein, respectively.

### In situ single-pass intestinal permeability study

**Figure 5:**
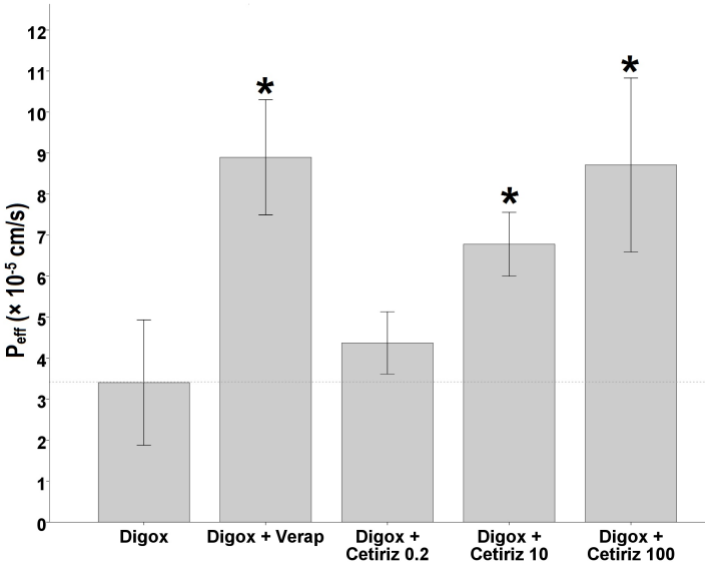
Effects of verapamil and 0.2, 10, and 100 µM cetirizine on the effective permeability (P_eff_) of digoxin. Bars show mean ± SD of at least three measurements. * p < 0.05 was considered as significance level.

To further conﬁrming the inhibitory effect of cetirizine on P-gp activity, the *in situ* experiments were conducted. For this purpose digoxin intestinal permeability, as a typical P-gp substrate, in jejunal segment of rats was determined. As shown in Figure 5, 0.2 µM cetirizine did not signiﬁcantly increase the P_eff_ of digoxin relative to control group (digoxin alone) (p > 0.05), however the difference reached to signiﬁcance level at higher concentrations (10 and 100 µM, p < 0.01). The P_eff_ values of digoxin (20 µM) in the absence and presence of verapamil, as a typical inhibitor, were 3.4 ± 0.8 and 8.9 ± 0.7 ×10^-5^cm/s, respectively. Whereas, the P_eff_ values of digoxin in the presence of 0.2, 10, and 100 µM cetirizine were found to be 4.4 ± 0.4, 6.8 ± 0.4, and 8.7 ± 1.0 ×10^-5^ cm/s, respectively.

**Figure 6:**
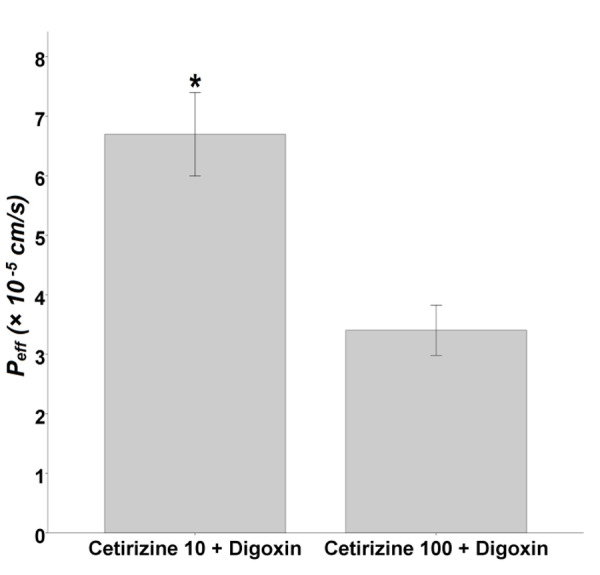
The effective permeability values of 10 and 100 µM cetirizine in the presence of 20 µM digoxin (n = 3). Bars show mean ± SD of at least three measurements. * p < 0.05 was considered as significance level.

The concentration of cetirizine was also determined in intestinal perfused samples, and P_eff_ values of 10 and 100 µM cetirizine were also calculated. The results, illustrated in Figure 6, showed that by increasing the concentration of cetirizine from 10 to 100 µM, the P_eff_ value decreased from 6.7 ± 0.7 to 3.4 ± 0.4 ×10^-5^cm/s. The difference between two groups was statistically significant (p = 0.02).

### Western Blotting 

**Figure 7:**
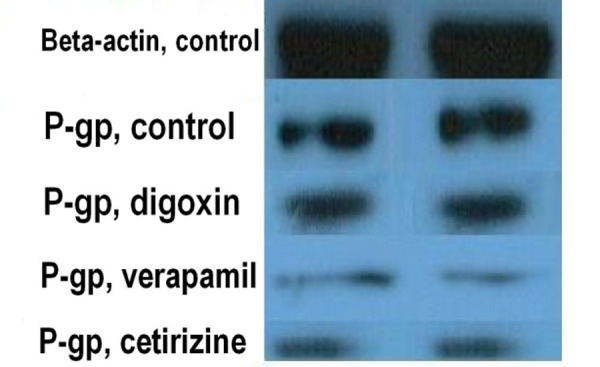
Western blot of P-gp and β- actin in 100 µM cetirizine and 300 µM verapamil treated, and control (non-treated) Caco-2 cells (bands of 2 samples of each are shown).

Immuno-blotting of Caco-2 cells were carried out using monoclonal antibody against P-gp, to further investigate the inhibitory effect of cetirizine treatment on P-gp activity after 48 h treating. The beta- actin protein expression was considered as internal immuno-blotting control. P-gp expression was presented as the ratio of P-gp band intensity to β -actin band intensity (P-gp/ β -actin) and were compared with verapamil and control bands in the same blot run.^14^ Low density of immuno-blot bands of cetirizine treated cells compared with those of untreated cells (control), as shown in Figure 7, demonstrated low expression of P-gp.

## Discussion

*In vitro* and *in situ* results of the present study showed P-gp inhibition potential of cetirizine. The mean intracellular concentration of Rho123 in 100 µM cetirizine treated Caco-2 cells was 1.8 times the control cells and was statistically significant (p < 0.05) while it was 8.4 times in verapamil treated cells (p < 0.01). In SPIP study, P_eff_ of digoxin was increased to 1.3, 2, and 2.6 times the control in the presence of 0.2, 10, and 100 µM cetirizine, respectively. Although, the difference was not significant for 0.2 µM concentrations, it reached to a high significant level at 10 and 100 µM cetirizine (p < 0.01). The upward trend of P_eff_ of digoxin showed a dose-dependent P-gp inhibition effect of cetirizine, at least in the range of 0.2-100 µM concentrations. In our study, low density of P-gp immune-blot bands of cetirizine treated Caco-2 cells relative to non-treated cells showed down-regulation of P-gp expression by cetirizine.

P-gp inhibitors, as demonstrated by photo-affinity labeling, may exert their inhibitory effect by one of the following three mechanisms: (1) altering P-gp expression; (2) Disrupting the ATP hydrolysis; and (3) competition for a binding site or reversible inhibition.^19^ It might be speculated that the P-gp inhibitory mechanisms of cetirizine are both altering P-gp expression and competition for P-gp binding sites. Western blotting results of present study confirm the idea. On the other hand, previous studies demonstrated P-gp substrate role of the cetirizine in mice blood-brain barrier (BBB),^20^ 1b (mdr1a/b) knockout mice,^21^ human MDR1-transfected Madin-Darby canine kidney cells,^21^ and human Caco-2 cells^22^ using monolayer efflux assay^20^ and other *in vitro* and *in situ* assays. To the best of our knowledge, both digoxin and cetirizine are substrates of P-gp and competition for binding sites of P-gp molecules by them is suggested. As shown in Figure 5, by increasing the concentration of cetirizine from 0.2 to 100 µM, digoxin intestinal P_eff_ raised from 4.4 ± 0.4 to 8.7 ± 1.0 ×10^-5^cm/s, but P_eff_ of cetirizine decreased from 6.7 ± 0.7 to 3.4 ± 0.4 ×10^-5^cm/s; this may confirm the competition of two drugs for P-gp binding sites.

From the literature review, the physicochemical properties of cetirizine may help to suggest the P-gp inhibition effect of cetirizine. Ekins *et al.* focused on identifying functional groups of P-gp inhibitors active molecules in their study. The study was carried out with 27 inhibitors of digoxin transport in Caco-2 cells *in vitro*. They found that two hydrophobic groups along with a hydrogen-bond acceptor group and an aromatic core were required for P-gp inhibition.^23^ The chemical structure of cetirizine was shown in Figure 1 (C_21_H_27_Cl_3_N_2_O_3_) and it has one hydrogen bond donor, 5 hydrogen bond acceptors, and 8 rotatable bonds. Log octanol/water partition coefficients, log P and log D, of cetirizine are 4.48 and 1.04 (pH = 7.4), respectively. Polli *et al*. studied the influence of P-gp on the brain concentrations of cetirizine and hydroxyzine. They concluded that P-gp influences the brain concentration of cetirizine, as a P-gp substrate, but not hydroxyzine. They investigated and compared the physicochemical properties of cetirizine and hydroxyzine and suggested that the difference in the substrate activity is related to the log D_oct_ (pH = 7.4) values. The log D_oct_, which is a measure of lipophilicity at pH = 7.4, for cetirizine was 1.04 compared with a value of 2.87 for hydroxyzine.^20^ The carboxylic group of cetirizine can interact with the basic nitrogen via folded conformers due to the molecular structure of cetirizine. This can cause relatively high lipophilicity at physiological pH. Oral bioavailability of cetirizine is more than 70% and shows high intestinal absorption in human.^7,24^

P-gp activity undoubtedly plays an important role in limiting intestinal permeability of BCS class II–IV P-gp substrates (not for BCS class I compounds). The high P-gp expression levels in the intestine make the moderately absorbed P-gp substrates more susceptible to P-gp-mediated efflux. Inhibition of P-gp by inhibitors, like cetirizine, can move compounds of BCS class III and IV (for example, digoxin) to class I and II, respectively, by significantly increasing the permeability.^25^ This may result in unexpected toxic effects of administrated drug(s). The unwanted drug-drug interactions may be reduced by considering the P-gp inhibitory effect or substrate activity of the concomitantly used drugs.

## Conclusion

In conclusion, our results demonstrated that cetirizine treatment could down-regulate the function and expression of P-glycoprotein *in vitro* and *in situ* and its inhibition activity is dose-dependent. The P-gp inhibitory effect of cetirizine must be considered for predicting potential drug–drug interactions when the drug is administered concurrently with P-gp substrate drugs. However further investigations with larger sample sizes are required to approve the obtained results and also find out the exact P-gp inhibitory mechanisms of cetirizine.

## Acknowledgments

We would like to thank the members of Drug Applied Research Center and Immunology Research Center of Tabriz University of Medical Sciences. This work was financially supported by Drug Applied Research Center of Tabriz University of Medical Sciences (Grant No. 91-123) and is a part of the PhD thesis of Dr. Mehran Mesgari Abbasi.

## Ethical Issues

Not applicable.

## Conflict of Interest

The authors report no conflicts of interest.

## References

[1] Kumar YS, Adukondalu D, Sathish D, Vishnu YV, Ramesh G, Latha AB (2010). P-glycoprotein- and cytochrome p-450-mediated herbal drug interactions. Drug Metabol Drug Interact.

[2] Kobori T, Harada S, Nakamoto K, Tokuyama S (2013). Functional alterations of intestinal p-glycoprotein under diabetic conditions. Biol Pharm Bull.

[3] Li Y, Huang L, Zeng X, Zhong G, Ying M, Huang M (2014). Down-regulation of P-gp expression and function after Mulberroside A treatment: potential role of protein kinase C and NF-kappa B. Chem Biol Interact.

[4] Lin JH (2007). Transporter-mediated drug interactions: Clinical implications and in vitro assessment. Expert Opin Drug Metab Toxicol.

[5] Horn JR, Hansten PD (2004). Drug interactions with digoxin: The role of P-glycoprotein. Pharm Times.

[6] Wessler JD, Grip LT, Mendell J, Giugliano RP (2013). The P-glycoprotein transport system and cardiovascular drugs. J Am Coll Cardiol.

[7] Chen C (2008). Physicochemical, pharmacological and pharmacokinetic properties of the zwitterionic antihistamines cetirizine and levocetirizine. Curr Med Chem.

[8] Bhatia NM, Ganbavale SK, Bhatia MS, More HN, Kokil SU (2008). Rp-HPLC and spectrophotometric estimation of ambroxol hydrochloride and cetirizine hydrochloride in combined dosage form. Indian J Pharm Sci.

[9] Souri E, Hatami A, Shabani Ravari N, Alvandifar F, Barazandeh Tehrani M (2013). Validating a stability indicating HPLC method for kinetic study of cetirizine degradation in acidic and oxidative conditions. Iran J Pham Res.

[10] Yu H, Cornett C, Larsen J, Hansen SH (2010). Reaction between drug substances and pharmaceutical excipients: Formation of esters between cetirizine and polyols. J Pharm Biomed Anal.

[11] Zrieki A, Farinotti R, Buyse M (2010). Cyclooxygenase-2 inhibitors prevent trinitrobenzene sulfonic acid-induced P-glycoprotein up-regulation in vitro and in vivo. Eur J Pharmacol.

[12] Zastre J, Jackson J, Bajwa M, Liggins R, Iqbal F, Burt H (2002). Enhanced cellular accumulation of a P-glycoprotein substrate, rhodamine-123, by Caco-2 cells using low molecular weight methoxypolyethylene glycol-block-polycaprolactone diblock copolymers. Eur J Pharm Biopharm.

[13] Mohammadzadeh R, Baradaran B, Valizadeh H, Yousefi B, Zakeri-Milani P (2014). Reduced ABCB1 expression and activity in the presence of acrylic copolymers. Adv Pharm Bull.

[14] Wang XD, Meng MX, Gao LB, Liu T, Xu Q, Zeng S (2009). Permeation of astilbin and taxifolin in Caco-2 cell and their effects on the P-gp. Int J Pharm.

[15] Patel JR, Barve KH (2012). Intestinal permeability of lamivudine using single pass intestinal perfusion. Indian J Pharm Sci.

[16] Zakeri-Milani P, Valizadeh H, Tajerzadeh H, Azarmi Y, Islambolchilar Z, Barzegar S (2007). Predicting human intestinal permeability using single-pass intestinal perfusion in rat. J Pharm Pharm Sci.

[17] Valizadeh H, Mehtari M, Zakeri-Milani P (2012). Evidence for enhanced intestinal absorption of digoxin by P-glycoprotein inhibitors. Trop J Pharm Res.

[18] Prasad N, Bhasker S (2012). Characterization of intestinal transport of vincristine in rats applying in situ single pass intestinal perfusion. Pharmacologia.

[19] Lopez D, Martinez-Luis S (2014). Marine natural products with P-glycoprotein inhibitor properties. Mar Drugs.

[20] Polli JW, Baughman TM, Humphreys JE, Jordan KH, Mote AL, Salisbury JA (2003). P-glycoprotein influences the brain concentrations of cetirizine (zyrtec), a second-generation non-sedating antihistamine. J Pharm Sci.

[21] Chen C, Hanson E, Watson JW, Lee JS (2003). P-glycoprotein limits the brain penetration of nonsedating but not sedating H1-antagonists. Drug Metab Dispos.

[22] Crowe A, Wright C (2012). The impact of P-glycoprotein mediated efflux on absorption of 11 sedating and less-sedating antihistamines using Caco-2 monolayers. Xenobiotica.

[23] Ekins S, Kim RB, Leake BF, Dantzig AH, Schuetz EG, Lan LB (2002). Application of three-dimensional quantitative structure-activity relationships of P-glycoprotein inhibitors and substrates. Mol Pharmacol.

[24] Zhang L, Cheng L, Hong J (2013). The clinical use of cetirizine in the treatment of allergic rhinitis. Pharmacol.

[25] Varma MV, Panchagnula R (2005). Prediction of in vivo intestinal absorption enhancement on P-glycoprotein inhibition, from rat in situ permeability. J Pharm Sci.

